# Morusin Functions as a Lipogenesis Inhibitor as Well as a Lipolysis Stimulator in Differentiated 3T3-L1 and Primary Adipocytes

**DOI:** 10.3390/molecules23082004

**Published:** 2018-08-10

**Authors:** Mi Rim Lee, Ji Eun Kim, Jun Young Choi, Jin Ju Park, Hye Ryeong Kim, Bo Ram Song, Ji Won Park, Mi Ju Kang, Young Whan Choi, Kyung Mi Kim, Dae Youn Hwang

**Affiliations:** 1 Department of Biomaterials Science, College of Natural Resources & Life Science/Life and Industry Convergence Research Institute, Pusan National University, Miryang 627-706, Korea; rlovemirim@naver.com (M.R.L.); prettyjiunx@naver.com (J.E.K.); junyoung4113@naver.com (J.Y.C.); jjpearl0005@naver.com (J.J.P.); hyeryeong.kim@kitox.re.kr (H.R.K.); 94sbr@naver.com (B.R.S.); pjw08260824@naver.com (J.W.P.); beautifulbead@naver.com (M.J.K.); 2 Department of Horticultural Bioscience, College of Natural Resources & Life Science, Pusan National University, Miryang 50463, Korea; ywchoi@pusan.ac.kr; 3 Novarex Co., Chungju 28126, Korea; kkm3507@novarex.co.kr

**Keywords:** morusin, lipolysis, lipogenesis, MDI, cell cycle, 3T3-L1

## Abstract

Conflicting results for morusin activity during adipogenic differentiation are reported in 3T3-L1 adipocytes and cancer cells. To elucidate the influence of morusin on fat metabolism, their anti-obesity effects and molecular mechanism were investigated in 3T3-L1 cells and primary adipocytes. Morusin at a dose of less than 20 µM does not induce any significant change in the viability of 3T3-L1 adipocytes. The accumulation of intracellular lipid droplets in 3T3-L1 adipocytes stimulated with 0.5 mM 3-isobutyl-1-methylxanthine, 1 µM dexamethasone, 10 µg/mL insulin in DMEM containing 10% FBS (MDI)-significantly reduces in a dose-dependent manner after morusin treatment. The phosphorylation level of members in the MAP kinase signaling pathway under the insulin receptor downstream also decrease significantly in the MDI + morusin-treated group compared to MDI + vehicle-treated group. Also, the expression of adipogenic transcription factors (*PPARγ* and *C/EBPα*) and lipogenic proteins (*aP2* and *FAS*) are significantly attenuated by exposure to the compound in MDI-stimulated 3T3-L1 adipocytes. Furthermore, the decrease in the G0/G1 arrest of cell cycle after culturing in MDI medium was dramatically recovered after co-culturing in MDI + 20 µM morusin. Moreover, morusin treatment induces glycerol release in the primary adipocytes of SD rats and enhances lipolytic protein expression (HSL, ATGL, and perilipin) in differentiated 3T3-L1 adipocytes. Overall, the results of the present study provide strong evidence that morusin inhibits adipogenesis by regulating the insulin receptor signaling, cell cycle and adipogenic protein expression as well as stimulating lipolysis by enhancing glycerol release and lipolytic proteins expression.

## 1. Introduction

Morusin is a well-known prenylated flavonoid purified from the root bark of *Morus austrakis* [1,2] and branch bark of *Ramulus mori* [3]. This compound has various biological activities such as anti-tumor, anti-inflammatory, anti-oxidant, anti-obesity, and anti-microbial, although most studies have focused on the anti-tumor effects [4]. Morusin dramatically suppresses the growth of different tumor types including breast cancer [5], glioblastoma [6], pancreatic cancer [7], hepatocarcinoma [8], prostate cancer [9], and gastric cancer [10]. Also, it inhibits the inflammatory response by suppressing the *COX* activity and *iNOS* expression [11], while strong antibacterial activity against Gram-positive bacteria was detected after morusin treatment [12]. However, very little evidence has reported the possibility that the biological role of morusin correlates with lipid metabolism.

Previous studies have shown contradictory results regarding the regulation of lipid metabolism by morusin. One study provided a clue to the possibility that morusin has anti-obesity activity. This compound was isolated as one of bioactive compounds from *Morus alba* var. *multicaulis* and inhibited the TG content and glycerol-3-phosphate dehydrogenase (DPDH) activity in MDI-stimulated 3T3-L1 adipocytes at 20 µM concentration [13]. However, another study showed that morusin stimulates lipid accumulation in cancer cells. The treatment of morusin induced the dose-dependent increase of lipid droplets in glioblastoma stem cells (GSCs) and enhanced the levels of adipogenic proteins including PPARγ, adipsin, aP2, and perilipin [6]. Furthermore, a similar effect on the accumulation of lipid droplets was observed in breast cancer cells of murine and human origin. The expression level of two adipogenic factors (C/EBP and PPARγ) and adipogenic proteins (adipsin D and perilipin) increased in morusin-treated MCF-7 cells [5]. These results, however, did not present a clear mechanism of action for the function of morusin in lipid metabolism. Therefore, further research was necessary to provide scientific evidence for these arguments.

In this study, we investigated the adipogenic, lipogenic, and lipolytic activity of morusin in MDI-stimulated 3T3-L1 and primary adipocytes from SD rats by analyzing the lipid accumulation, lipogenic factors, cell cycle and lipolytic factors. Our results provide additional scientific evidence that morusin suppresses adipogenesis and lipogenesis in MDI-stimulated 3T3-L1 cells and stimulates lipolysis in the primary adipocytes of SD rats. 

## 2. Materials and Methods

### 2.1. Cell Culture and Adipocyte Differentiation

3T3-L1 preadipocytes have the potential to differentiate into adipocyte-like phenotypes; the cells were obtained from the American Type Culture Collection (Mannassas, VA, USA). Cells were cultured in Dulbecco Modified Eagle’s Medium (DMEM, Welgene, Gyeongsan-si, Korea) supplemented with 10% fetal bovine serum (FBS, Welgene, Gyeongsan-si, Korea), l-glutamine, penicillin, and streptomycin (Thermo Scientific, Waltham, MA, USA), in a humidified incubator at 37 °C under 5% CO_2_ and 95% fresh air. 

**Figure 1:**
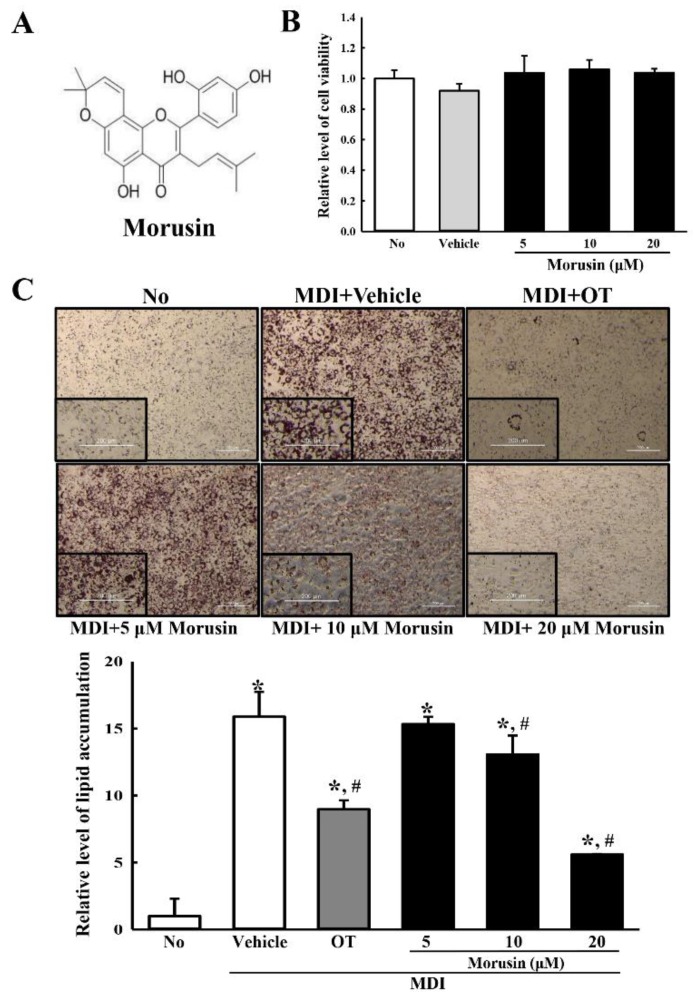
Chemical structure and cytotoxicity of morusin. (**A**) Chemical structure of morusin; (**B**) Viability of 3T3-L1 adipocytes to morusin. After incubation with morusin for 24 h, the cell viability was determined by the MTT assay in triplicate; (**C**) Analysis of lipid accumulation. The 3T3-L1 adipocytes were cultured in MDI medium with three different concentration of morusin for eight days, after which they were subjected to Oil Red O staining analysis. The images of the Oil Red O stained cells were observed with an inverted microscope at 100× magnification. The level of the stained lipid droplets was quantified by the absorbance at 510 nm using a Molecular Devices VERSA max Plate reader. The data represents the means ± SD of three replicates. * indicates *p* < 0.05 compared to the untreated group. ^#^ indicates *p* < 0.05 compared to the MDI + vehicle-treated group. OT; orlistat, MDI; adipogenic cocktail consisting of 3-isobutyl-1-methylxanthine, dexamethasone, and insulin.

Differentiation of 3T3-L1 preadipocytes was induced following a previously described method [14]. Briefly, cells were grown to more than 80–90% confluence (differentiation day 0); normal media was then replaced with differentiation medium (MDI) containing 3-isobutyl-1-methylxanthine (0.5 mM), dexamethasone (1 μM) and insulin (5 μg/mL) in DMEM supplemented with 10% FBS. After two days (differentiation day 2), cells were maintained in DMEM supplemented with 10% FBS and 5 μg/mL insulin for two more days (differentiation day 4), followed by culturing for an additional four days in DMEM supplemented with 10% FBS (differentiation day 8). Morusin (Toronto Research Chemicals, North York, ON, Canada) (Figure 1) was added to the medium at three different concentration (5, 10 and 20 µM) throughout the entire culture period (differentiation day 0 to day 8). 

### 2.2. Cell Viability Assay

Cell viability was determined using the tetrazolium compound 3-[4,5-dimethylthiazol-2-yl]-2,5-diphenyltetrazolium bromide (MTT) assay (Sigma-Aldrich Co., St. Louis, MO, USA). To determine the cell viability, 3T3-L1 cells were seeded at a density of 1 × 10^4^ cells/0.2 mL and grown for 24 h in a 37 °C incubator. When the cells attained 70–80% confluence, they were treated with vehicle (DMSO), and exposed to 5, 10, and 20 μM of morusin. Following incubation for 24 h, the supernatants of the 3T3-L1 cells were discarded, after which 0.2 mL of fresh DMEM media and 50 µL of MTT solution (2 mg/mL in PBS) were added to each well. Cells were then incubated at 37 °C for 4 h, after which the formazan precipitate was dissolved in DMSO and the absorbance was read at 570 nm using a VERSA max Plate reader (Molecular Devices, Sunnyvale, CA, USA).

### 2.3. Oil Red-O Staining

Lipid accumulation was detected in 3T3-L1 cells after staining with Oil Red-O dye, as described in previous reports [7]. Briefly, cells were fixed with 4% formaldehyde for 60 min and washed three times with distilled water, after which they were incubated with 0.5% Oil red-O dye (Sigma-Aldrich Co.) in 100% isopropanol for 30 min at room temperature. After washing three times with distilled water, the stained fat droplets in the adipocytes were observed microscopically at 100× magnification (Leica Microsystems, Wetzlar, Germany). Spectrophotometric analysis of the stain was performed by dissolving the stained lipid droplets in isopropanol. Finally, the absorbance was measured at 510 nm using a Molecular Devices VERSA max Plate reader.

### 2.4. Care and Use of Laboratory Animals

The protocol for animal experiments was reviewed and approved by the Pusan National University Institutional Animal Care and Use Committee (PNU-IACUC; Approval Number PNU-2017-1461). All SD rats were handled at the Pusan National University-Laboratory Animal Resources Center, which is accredited by the Korea Food and Drug Administration (FDA) (Accredited Unit Number-000231) and AAALAC International (Accredited Unit Number; 001525). Eight-week-old male SD rats were purchased from Samtako BioKorea Inc. (Osan, Korea) and provided with ad libitum access to water and a standard irradiated chow diet (Samtako BioKorea Inc.). During the experiment, rats were maintained in a specific pathogen-free state (SPF) under a strict light cycle (lights on at 08:00 and off at 20:00) at 23 ± 2 °C and 50 ± 10% relative humidity.

### 2.5. Isolation and Culture of Primary Adipocytes

Briefly, the adult male SD rats were sacrificed using CO_2_ gas, and the intra-abdominal adipose tissues were then dissected. The tissues (30 g) were minced in 5 mL of DMEM supplemented with 1 mg/mL type I collagenase (Worthington Biochemical Co., Freehold, NJ, USA) and 1% BSA (MP Biomedicals, Illkirch, France), and subsequently incubated at 37 °C for 30 min in a shaking incubator (JSR, Gongju-City, Korea). The homogenate of the minced adipose tissue was filtered through a 100 µm nylon mesh and washed three times in KRH (Krebs ringer/HEPES solution: 25 mM NaHCO_3_, 125 mM NaCl, 5 mM glucose, 2.5 mM KCl, 1.25 mM NaH_2_PO_4_, 2 mM CaCl_2_, 1 mM MgCl_2_, 25 mM HEPES) containing 1% BSA. Finally, after centrifugation, the pellets of the harvested adipocytes were re-suspended in KRH containing 3% BSA. Primary adipocytes were cultured in KRH supplemented with 3% BSA and maintained in a humidified incubator at 37 °C under 5% CO_2_ and 95% air. Thereafter, the adipocytes were seeded onto 24-well plates for each experimental protocol and incubated with different concentration of Morusin to measure the release of free glycerol.

### 2.6. Real Time-PCR Analysis

The mRNA levels of *PPARγ*, *C/EBPα*, *aP2* and *FAS* were measured by RT-PCR as previously described [15]. Briefly, total RNA molecules were purified from the cultured cells using RNAzol (Tel-Test Inc., Friendswood, TX, USA). After quantification of RNA using a NanoDrop system (Biospec-nano, Shimadzu Biotech, Kyoto, Japan), the complement DNA (cDNA) was synthesized using a mixture of total RNA (5 µg), oligo-dT primer (Invitrogen, Carlsbad, CA, USA), dNTP and reverse transcriptase (Superscript II, 18064-014, Invitrogen, 200 U/µL). Q-PCR was conducted with a cDNA template and 2× Power SYBR Green (TOYOBO Co., Osaka, Japan) using the following cycles: 15 s at 95 °C, 30 s at 55 °C, and 60 s at 70 °C. The primer sequences for target gene expression identification were as follows: *PPARγ*, sense primer: 5′-GAG TTC ATG CTT GTG AAG GAT GCA AGG-3′, anti-sense primer: 5′-CAT ACT CTG TGA TCT CTT GCA CG-3′; *C/EBPα*, sense primer: 5′-GGA ATC TCC TAG TCC TGG CTT GC-3′, anti-sense primer: 5′-GGA ATC TCC TAG TCC TGG CTT GC-3′; *aP2*, sense primer: 5′-GAA CCT GGA AGC TTG TCT CCA GTG-3′, anti-sense primer: 5′-GAT GCT CTT CAC CTT CCT GTC GTC TGC-3′; *FAS*, sense primer: 5′-GAT CCT GGA ACG AGA ACA CGA TCT GG-3′, anti-sense primer: 5′-GAT CCT GGA ACG AGA ACA CGA TCT GG-3′, anti-sense primer: 5’-AGA CTG TGG AAC ACG GTG GTG GAA CC-3′; *β-actin* sense primer: 5′-GTG GGG CGC CCC AGG CAC CAG GGC -3′, anti-sense primer: 5′-CTC CTT AAT GCT ACG CAC GAT TTC-3′. The reaction cycle during which PCR products exceeded this fluorescence intensity threshold during the exponential phase of the PCR amplification was considered the threshold cycle (Cq). Following Livak and Schmittgen’s method, the expression of the target gene was quantified relative to that of the housekeeping gene β-actin, based on a comparison of the Cqs at a constant fluorescence intensity [16]. 

### 2.7. Western Blot

For Western blot assay, total protein of 3T3-L1 cells was extracted using the Pro-Prep Protein Extraction Solution (iNtRON Biotechnology, Seongnam, Korea), followed by quantification using a SMARTTM BCA Protein Assay Kit (Thermo Scientific, Waltham, MA, USA). Equal amounts of proteins (30 μg) were loaded and separated by 4–20% sodium dodecyl sulfate–polyacrylamide gel electrophoresis (SDS-PAGE) for 2 h, after which the resolved proteins were transferred to nitrocellulose membranes for 2 h at 40 V. Each membrane was then incubated separately overnight at 4 °C with the following primary antibodies, all procured from Cell Signaling Technology, Danvers, MA, USA and diluted 1:1000: anti-perilipin antibody (#9349S), anti-p-perilipin antibody (#9621S), anti-HSL antibody (#4107S), anti-p-HSL antibody (#4139S), anti-ATGL antibody (#2183S) and anti-β-actin antibodies (#4967S, diluted 1:3000). The probed membranes were then washed with washing buffer (137 mM NaCl, 2.7 mM KCl, 10 mM Na_2_HPO_4_, and 0.05% Tween 20) and incubated with 1:1000 diluted horseradish peroxidase (HRP)-conjugated goat anti-rabbit IgG (Invitrogen, Carlsbad, CA, USA) at room temperature for 1 h. Finally, the membrane blots were developed using Amersham ECL Select Western Blotting detection reagent (GE Healthcare, Little Chalfont, UK). The chemiluminescence signals that originated from specific bands were detected using FluorChemi^®^FC2 (Alpha Innotech Co., San Leandro, CA, USA).

### 2.8. Cell Cycle Assay

The cell cycle was evaluated using a Muse™Cell Cycle Kit (MCH100106, Millipore Co., Billerica, MA, USA) according to the manufacturer’s instructions. Briefly, 3T3-L1 cells were cultured in 100-mm^2^ dishes (3 × 10^5^ cells/dish), then treated with MDI and three different concentrations of Morusin (5, 10 and 20 μM) for 24 h. Total cells from subset groups were harvested by centrifugation at 3000× *g* for 5 min and fixed with 70% EtOH at −20 °C for 3 h. The fixed cells were washed with 1× PBS and resuspended in 200 μL of Cell Cycle Reagent. Following incubation at 37 °C in a CO_2_ incubator for 30 min, cell cycles were analyzed using FACS (Millipore Co., Billerica, MA, USA).

### 2.9. Measurement of Free Glycerol Release

Free glycerol release from adipocytes was measured using the free glycerol reagent (Sigma-Aldrich Co., St. Louis, MO, USA) according to the manufacturer’s protocols. To measure the glycerol level, adipocytes were seeded at a density of 2 × 10^5^ cells/mL in KRH and cultured in a 37 °C incubator. After 24 h, they were either untreated, treated with vehicle (dH_2_O or DMSO), or pretreated with 10 μM of isoproterenol hydrochloride (standard compound; Sigma-Aldrich Co.) or 5, 10 and 20 μM of morusin. Following incubation for 24 h, the culture medium was collected from the primary adipocytes treated with morusin and heated at 65 °C for 15 min to inactivate any enzymes released by the adipocytes. The inactivated medium (10 μL) was then mixed with 200 μL of glycerol detection reagent, after which the absorbance was read at 570 nm using a Vmax plate reader (Molecular Devices, Sunnyvale, CA, USA).

### 2.10. Statistical Analysis

Statistical significance was evaluated using one-way Analysis of Variance (ANOVA) (SPSS for Windows, Release 10.10, Standard Version, Chicago, IL, USA) followed by Tukey’s post hoc *t*-test for multiple comparison. All data were expressed as the means ± SD. A *p* value less than 0.05 was considered statistically significant.

## 3. Results

### 3.1. Cytotoxicity of Morusin

To evaluate the cytotoxicity of morusin against adipocytes, the cell viability of 3T3-L1 adipocytes was assessed following exposure to 5, 10 and 20 µM of morusin. All the treated groups maintained a constant level of viability when compared to the untreated or vehicle-treated groups. Also, cells retained their morphology after morusin treatment (Figure 1B). These results indicate that morusin does not induce any significant toxicity to 3T3-L1 adipocytes at a concentration less than 20 µM.

### 3.2. Inhibitory Effect of Morusin on Lipid Accumulation

**Figure 2:**
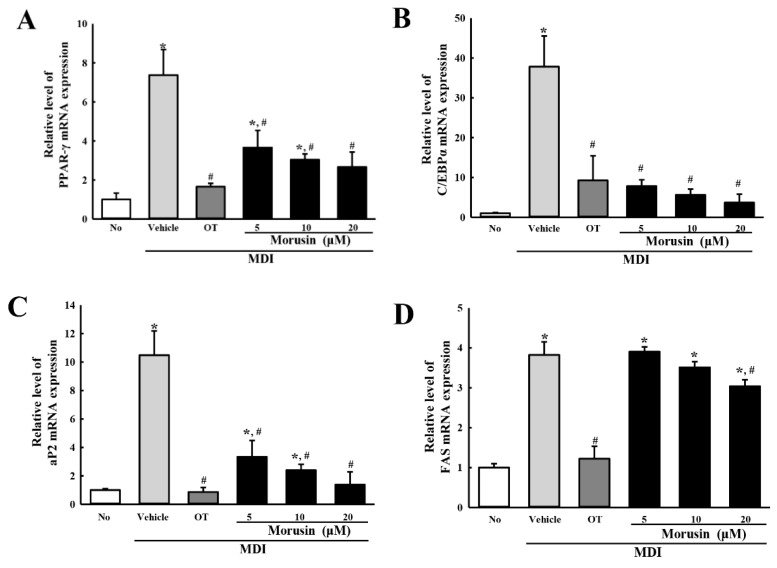
mRNA analysis of adipogenic and lipogenic factors. The mRNA levels of two adipogenic transcription factors (*PPARγ* (**A**) and *C/EBPα* (**B**)) and two lipogenic proteins (*aP2* (**C**) and *FAS* (**D**)) genes were measured by RT-PCR in the MDI + morusin-treated 3T3-L1 adipocytes using specific primers. The data represents the means ± SD of three replicates. * indicates *p* < 0.05 compared to the untreated group. ^#^ indicates *p* < 0.05 compared to the MDI + vehicle-treated group. OT; orlistat, MDI; adipogenic cocktail consisting of 3-isobutyl-1-methylxanthine, dexamethasone, and insulin.

To investigate the inhibitory effect of morusin on lipid accumulation, the intracellular lipid droplets were measured by oil red O staining in MDI-stimulated 3T3-L1 adipocytes treated with morusin for eight days. Oil red O-stained materials (OROSM) accumulation was observed as numerous large intracellular droplets after incubating mature 3T3-L1 adipocytes in a medium containing MDI. The number of droplets was dramatically enhanced as compared to the untreated group. However, the lipid accumulations significantly decreased in a dose-dependent manner after morusin co-treatment. The highest suppression of lipid droplets accumulation was observed in MDI + 20 μM morusin-treated group (Figure 2C). Taken together, these results indicate that morusin suppresses the increased accumulation of lipid droplets induced by culturing 3T3-L1 adipocytes in an MDI medium.

### 3.3. Suppression of Adipogenic Transcription Factors and Lipogenic Proteins after Morusin Exposure

To examine the inhibitory effect of morusin on the expression of adipogenic and lipogenic factors, the mRNA level of two transcription factors (*PPARγ* and *C/EBPα*) and two lipogenic proteins (*aP2* and *FAS*) were evaluated in the MDI-stimulated 3T3-L1 adipocytes after exposure to morusin. The mRNA expression of the two transcription factors was higher in the MDI + vehicle-treated group than in the untreated group, with a significant dose-dependent decrease observed in the MDI + morusin-treated group (Figure 2A,B). A similar response was observed in the mRNA expression of the two lipogenic proteins, although the decrease in *aP2* mRNA expression was higher than *FAS* mRNA (Figure 2C,D). These results suggest that morusin suppresses the increased mRNA expression of adipogenic transcription factors and lipogenic proteins induced when cultured in an MDI medium.

### 3.4. Effect of Morusin on the Insulin Receptor Downstream Signaling Pathway

**Figure 3:**
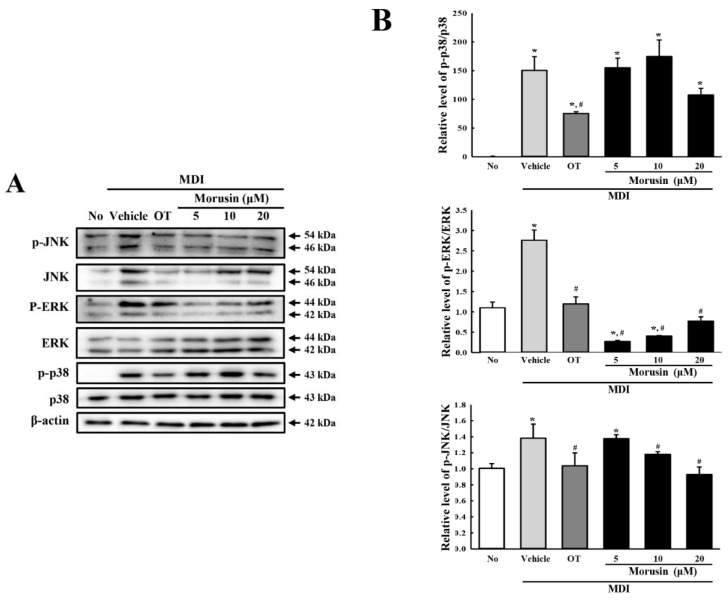
Expression analysis of three members of the MAPK signaling pathway. (**A**) After the incubation of 3T3-L1 adipocytes in MDI + morusin, the expression levels of JNK, ERK, p38 and β-actin were detected with specific antibodies, followed by horseradish peroxidase-conjugated goat anti-rabbit IgG. (**B**) Band intensities were measured by using an imaging densitometer, and the levels of each protein were calculated relative to the intensity of the actin bands. The data represents the means ± SD of three replicates. * indicates *p* < 0.05 compared to the untreated group. ^#^ indicates *p* < 0.05 compared to the MDI+vehicle-treated group. OT; orlistat, MDI; adipogenic cocktail consisting of 3-isobutyl-1-methylxanthine, dexamethasone, and insulin.

The MAPK signaling pathway has a key role in signal transmission from the insulin receptor to adipogenic transcription factors [17]. We therefore investigated whether the inhibition of adipogenic transcription factors is accompanied by alteration of the insulin receptor downstream signaling. To achieve this, we measured the expression of three members of the MAPK signaling pathway (p38, JNK and ERK) in MDI + morusin-treated 3T3-L1 adipocytes. Of the three members evaluated, p38 and JNK showed a similar pattern. Enhancement of phosphorylation by culturing in MDI medium significantly decreased in the MDI + 20 μM morusin-treated group compared with MDI + vehicle-treated group. However, the phosphorylation of ERK was remarkably lower, but not significant, in the MDI + morusin-treated group than in MDI + vehicle-treated group (Figure 3). The above results indicate that the effects of morusin on adipogenesis may be correlated with regulation of the MAPK signaling pathway under the downstream signaling of the insulin receptor. 

### 3.5. Effect of Morusin on the Regulation of the Cell Cycle

**Figure 4:**
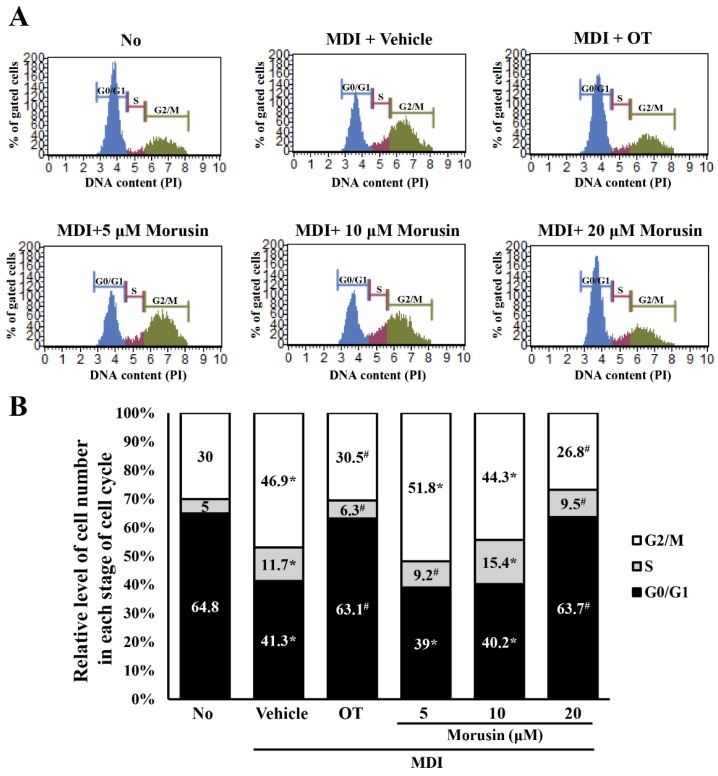
Cell cycle analysis of morusin-treated 3T3-L1 adipocytes. (**A**) The cell cycle distribution was determined by flow cytometric analysis of the DNA content of nuclei of cells after staining with propidium iodide. After treatment with morusin in MDI-stimulated adipocytes, (**B**) the number of cells in the G0/G1, S, and G2/M stage were determined at each time point. The data represents the means ± SD of three replicates. * indicates *p* < 0.05 compared to the untreated group. ^#^ indicates *p* < 0.05 compared to the MDI + vehicle-treated group. OT; orlistat, MDI; adipogenic cocktail consisting of 3-isobutyl-1-methylxanthine, dexamethasone, and insulin.

Culturing in MDI medium induces the progression of G0/G1 cell cycle arrest triggered by confluent culture of 3T3-L1 adipocytes [18]. To examine whether the suppressive effects of morusin on adipogenesis accompanies the regulation of cell cycle, the number of cells at each stage of the cell cycle were counted in the subset groups. In the MDI + vehicle-treated group, the cell number in the G0/G1 stage was significantly decreased, while those in the S and G2/M stage were enhanced. However, MDI + 20 μM morusin co-treatment induced recovery up to 98% of the number observed in the G0/G1 stage of the untreated group, while that in the G2/M stage was slightly decreased (Figure 4). These results suggested that morusin treatment restores the cell cycle arrest in the G0/G1 stage and stimulates progression from the G2/M stage to the G1 stage.

### 3.6. Effect of Morusin on Lipolysis

**Figure 5:**
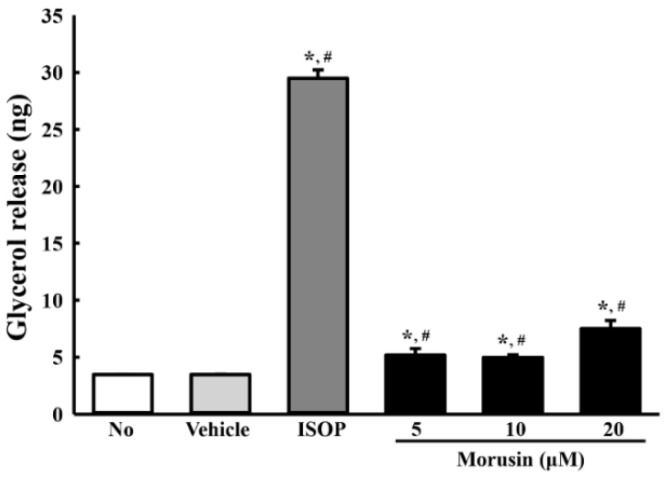
Free glycerol release of morusin-treated primary adipocytes. Released glycerol was measured in the supernatant of primary adipocytes treated with three different concentrations of morusin. The data represents the means ± SD of three replicates. * indicates *p* < 0.05 compared to the untreated group. ^#^ indicates *p* < 0.05 compared to the vehicle-treated group. ISOP; Isoproterenol.

**Figure 6:**
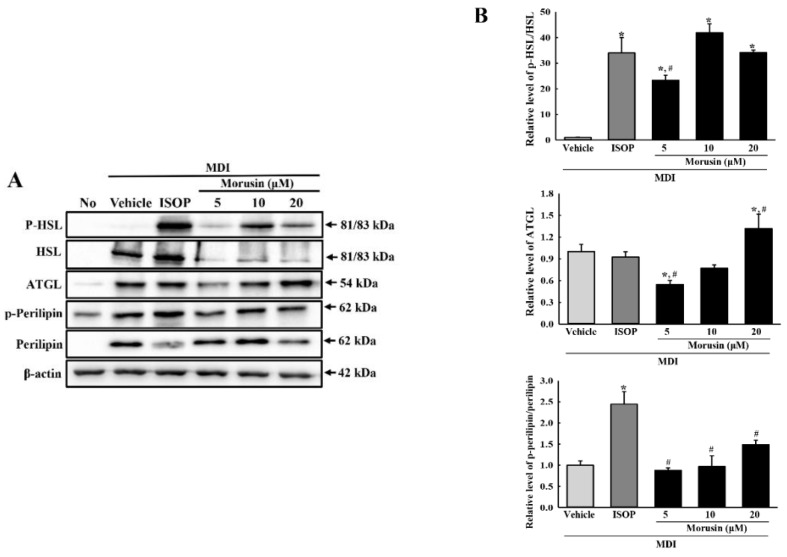
Expression analysis of lipolytic proteins. (**A**) After the incubation of MDI + morusin in 3T3-L1 adipocytes, the expression levels of HSL, ATGL, perilipin and β-actin were detected with specific antibodies, followed by horseradish peroxidase-conjugated goat anti-rabbit IgG. (**B**) Band intensities were measured using an imaging densitometer, and the relative levels of each protein was calculated relative to the intensity of actin bands. The data represents the means ± SD of three replicates. * indicates *p* < 0.05 compared to the untreated group. ^#^ indicates *p* < 0.05 compared to the MDI + vehicle-treated group. OT; orlistat, MDI; adipogenic cocktail consisting of 3-isobutyl-1-methylxanthine, dexamethasone, and insulin.

To verify the stimulatory effect of morusin on lipolysis, the levels of free glycerol and the expression level of lipolytic proteins were measured in the culture medium of primary adipocytes and MDI-stimulated 3T3-L1 cells after treatment with 5, 10 and 20 µM of morusin. Significantly enhanced levels of glycerol release were observed in morusin-treated primary adipocytes with the highest level measured in the MDI + 20 μM morusin-treated group (Figure 5). A similar alteration was observed in the expression of lipogenic proteins in 3T3-L1 adipocytes treated with morusin. Of the three lipogenic proteins studied, the phosphorylation level of HSL was dramatically increased in all MDI + morusin-treated groups as compared with MDI + vehicle-treated group. However, the phosphorylation level of perilipin and the expression level of ATGL were significantly increased in only the MDI + 20 μM morusin-treated group (Figure 6). Overall, these findings indicate that morusin promotes lipolysis of adipocytes derived from SD rats and 3T3-L1 adipocytes.

## 4. Discussion

Various compounds with lipolytic activity and anti-lipogenesis activity were isolated as potential drug candidates from herbal medicines and natural products used to prevent accumulation of body fat. To date, many studies report anti-obesity effects after the treatment of several natural compounds such as resveratrol [19], ramalin [20], zeaxanthin [21], and eupatilin [22]. In this study, we tried to obtain scientific evidences of the role of morusin on the inhibition of lipogenesis and stimulation of lipolysis in 3T3-L1 cells and primary adipocytes. Our results indicate that morusin prevents lipid accumulation in MDI-stimulated 3T3-L1 cells through the regulation of adipogenic transcription factors, lipogenic proteins during the early stage of adipocytes differentiation, and cell cycle. In addition, it stimulates glycerol release from the primary adipocytes and enhances the expression level of lipolytic proteins in differentiated 3T3-L1 adipocytes.

Two adipogenic transcription factors (*PPARγ* and *C/EBPα*) as well as lipogenic proteins (*aP2* and *FAS*) play a key role during adipogenesis namely, the differentiation of fibroblast-like preadipocytes into mature lipid accumulated, insulin-responsive adipocytes [23]. Especially, the expression of PPARγ and C/EBPα is enhanced during the intermediate stage of adipocyte differentiation [24]. Upregulated PPARγ, and C/EBPα induces the transcription of aP2 and FAS genes associated with producing and maintaining the adipocyte phenotypes in the late stage [25]. For this reason, previous studies have used them as distinct markers to identify novel compounds with anti-obesity activity. Although the expression levels of adipogenic transcription factors and lipogenic proteins are significantly increased in MDI-stimulated 3T3-L1 cells, these levels recovered after treatment with several single compounds isolated from natural products including resveratrol [19], ramalin [20], zeaxanthin [21], and eupatilin [22]. Results of this current study are consistent with previous studies; we observed a decrease of two adipogenic transcription factors and two lipogenic proteins in MDI-stimulated 3T3-L1 cells after morusin treatment. These results provide scientific evidence for the molecular mechanism of the anti-adipogenic effect of morusin on the differentiation and lipid accumulation of 3T3-L1 adipocyte.

The number of adipocytes at the early stages of adipogenesis increases with cell cycle progression during the induction of mitotic clonal expansion (MCE) [26]. Several anti-obesity compounds contribute to the regulation of cell cycle progression in MDI-stimulated 3T3-L1 cells. The decrease of G0/G1 arrested population in MDI-stimulated cells recovered after treatment with dioscin [27], ramalin [20], and sinigrin [28]. In addition, cell cycle progression is mediated by MAPK signaling pathway during adipogenesis [29]. The high level of phosphorylated p38, JNK and ERK in MDI-stimulated adipocytes significantly decreases after exposure to ramalin [20] and dioscin [27], while the suppression of ERK and p38 phosphorylation was observed in dioscin-treated adipocytes [27]. In this study, our data indicates that morusin treatment induces the recovery of the G0/G1 arrest of cell cycle progression and MAPK signaling pathway in MDI-stimulated 3T3-L1 adipocytes. Most results from the current study are very similar to previous studies, although few differences were detected in the recovery rate. Especially, the phosphorylation level of ERK is dose-dependently enhanced after morusin treatment, while previous results show a decreasing pattern. This contradiction may be due to different properties of each compound. Therefore, further studies are required to verify the reverse effects of morusin treatment on the phosphorylation of ERK.

Among the essential properties of an anti-obesity drug, lipogenic activity and lipolytic activity are considered very important. However, recent studies have focused on the lipogenic activity of single compounds isolated from medicinal plants and have not evaluated their lipolytic activity [7,22,27,28]. Only a few studies have reported the lipolytic activity of single compounds from herbal plants. Glycerol release and perilipin expression decrease in TNF-α-stimulated 3T3-L1 cells after resveratrol treatment [19]. The mRNA expression of perilipin is dose-dependently inhibited in MDI-stimulated 3T3-L1 after zeaxanthin treatment, while HSL mRNA remains constant [21]. In the current study, we examined the lipogenic activity and lipolytic activity of morusin in MDI-stimulated 3T3-L1 adipocytes to evaluate their anti-obesity effects. Our study shows that morusin stimulates lipolysis and inhibits lipogenesis in 3T3-L1 adipocytes.

Meanwhile, the free glycerol release and the expression of lipolytic proteins are considered as a major factor to evaluate the lipolytic activity of specific compounds [19,21]. Most studies measured the level of these factors in only 3T3-L1 adipocytes after the treatment of each compound [19,21], although the range was extensive. However, in our study, the free glycerol release was measured in the primary adipocytes derived from SD rats, while the expression of lipolytic proteins was detected in 3T3-L1 cells. The results of free glycerol release did not completely correlate with the alteration on the expression of lipolytic proteins in morusin-treated cells. These differences in the lipolytic effect of morusin can be attributed to the purity of primary adipocytes used in the analysis for glycerol release.

## 5. Conclusions

**Figure 7:**
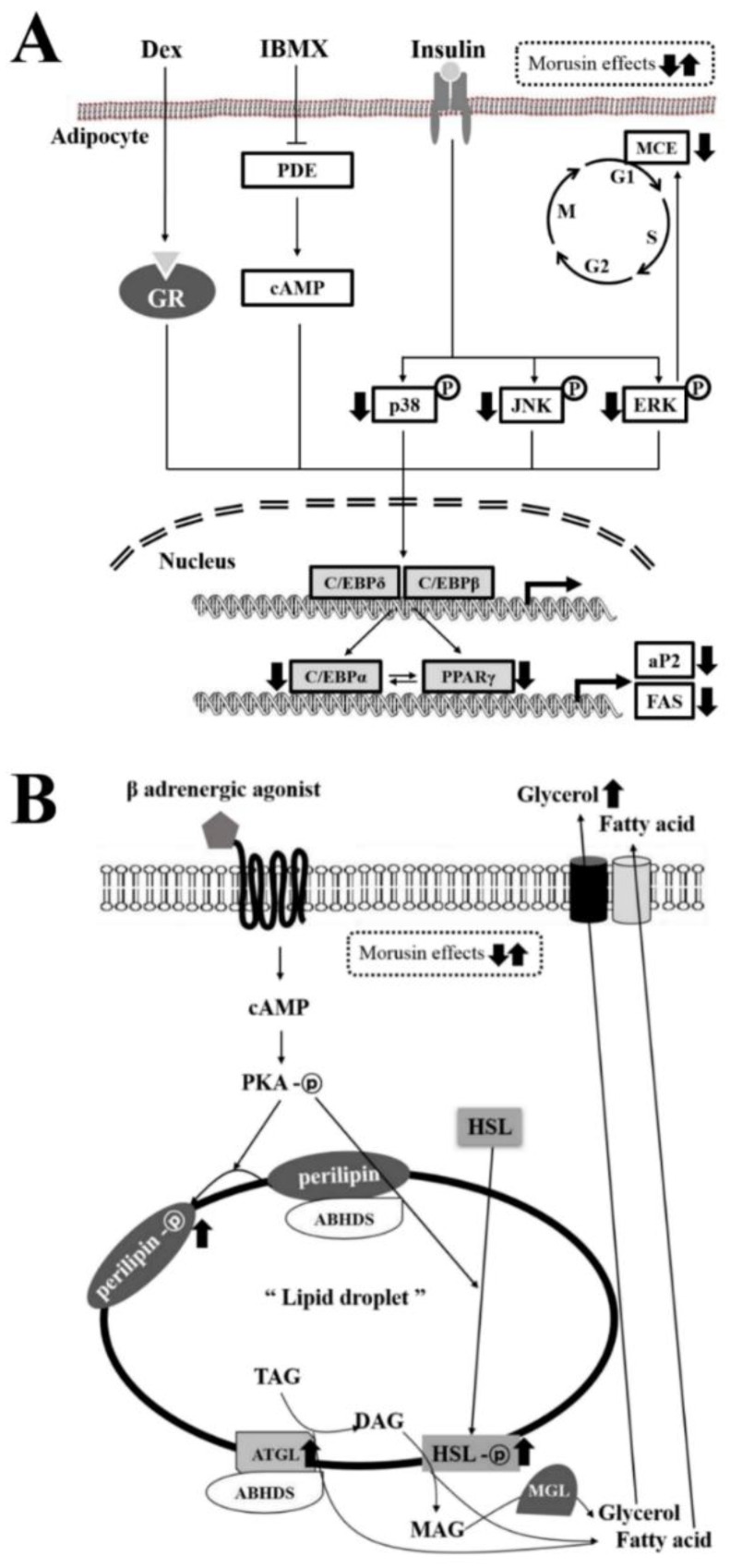
Suggested mechanism of action of morusin in MDI-stimulated 3T3-L1 cells and primary adipocytes. In this scheme, (**A**) anti-lipogenic effects of morusin are thought to be exerted via the inhibition of adipogenic transcription factors and lipogenic proteins, recovery of G0/G1 arrest in cell cycle, and the regulation of the MAPK signaling pathway. (**B**) Anti-lipolytic effects of morusin are thought to stimulate the activation of lipolytic proteins.

The results of the present study indicate that morusin inhibits lipogenesis through its effects on the expression of adipogenic factors, cell cycle arrest, and MAPK signaling pathway. Also, it stimulates lipolysis via the regulation of major lipid droplet-associated proteins in differentiated adipocytes (Figure 7A,B). Considering the above data, we postulate that morusin has the potential for use as a lipolytic agent for the treatment of obesity.

## References

[1] Ko H.H., Yu S.M., Ko F.N., Teng C.M., Lin C.N. (1997). Bioactive constituents of Morus australis and Broussonetia papyrifera. J. Nat. Prod..

[2] Ko H.H., Wang J.J., Lin H.C., Wang J.P., Lin C.N. (1999). Chemistry and biological activities of constituents from Morus australis. Biochim. Biophys. Acta.

[3] Wan L.Z., Ma B., Zhang Y.Q. (2014). Preparation of morusin from Ramulus ori and its effects on mice with transplanted H22 hepatocarcinoma. Biofactors.

[4] Xue J., Li R., Zhao X., Ma C., Lv X., Liu L., Liu P. (2018). Morusin induces paraptosis-like cell death through mitochondrial calcium overload and dysfunction in epithelial ovarian cancer. Chem. Biol. Interact..

[5] Li H., Wang Q., Dong L., Liu C., Sun Z., Gao L., Wang X. (2015). Morusin suppresses breast cancer cell growth in vitro and in vivo through C/EBPβ and PPARγ mediated lipoapoptosis. J. Exp. Clin. Cancer Res..

[6] Guo H., Liu C., Yang L., Dong L., Wang L., Wang Q., Li H., Zhang J., Lin P., Wang X. (2016). Morusin inhibits glioblastoma stem cell growth in vitro and in vivo through stemness attenuation, adipocyte transdifferentiation, and apoptosis induction. Mol. Carcinog..

[7] Kim C.W., Kim J.H., Oh E.Y., Nam D.W., Lee S.G., Lee J.H., Kim S.H., Sim B.S., Ahn K.S. (2016). Blockage of STAT3 signaling pathway by morusin induces apoptosis and inhibits invasion in human pancreatic tumor cells. Pancreas.

[8] Gao L., Wang L., Sun Z., Li H., Wang Q., Yi C., Wang X. (2017). Morusin shows potent antitumor activity for human hepatocellular carcinoma in vitro and in vivo through apoptosis induction and angiogenesis inhibition. Drug Des. Devel. Ther..

[9] Cho S.W., Na W.J., Choi M.J., Kang S.J., Lee S.G., Choi C.Y. (2017). Autophagy inhibits cell death induced by the anti-cancer drug morusin. Am. J. Cancer Res..

[10] Wang F., Zhang D., Mao J., Ke X.X., Zhang R., Yin C., Gao N., Cui H. (2017). Morusin inhibits cell proliferation and tumor growth by down-regulating c-Myc in human gastric cancer. Oncotarget.

[11] Bellik Y., Boukraâ L., Alzahrani H.A., Bakhotmah B.A., Abdellah F., Hammoudi S.M., Iguer-Ouada M. (2013). Molecular mechanism underlying anti-inflammatory and anti-allergic activities of phytochemicals: an update. Molecules.

[12] Sohn H.Y., Son K.H., Kwon C.S., Kwon G.S., Kang S.S. (2004). Antimicrobial and cytotoxic activity of 18 prenylated flavonoids isolated from medicinal plants: *Morus alba* L., *Morus mongolica* Schneider, *Broussnetia papyrifera* (L.) Vent, *Sophora flavescens* Ait and *Echinosophora koreensis* Nakai. Phytomedicine.

[13] Yang Z.G., Matsuzaki K., Takamatsu S., Kitanaka S. (2011). Inhibitory effects of constituents from *Morus alba* var. *multicaulis* on differentiation of 3T3-L1 cells and nitric oxide production in RAW264.7 cells. Molecules.

[14] Park H.J., Cho J.Y., Kim M.K., Koh P.O., Cho K.W., Kim C.H., Lee K.S., Chung B.Y., Kim G.S., Cho J.H. (2012). Anti-obesity effect of *Schisandra chinensis* in 3T3-L1 cells and high fat diet-induced obese rats. Food Chem..

[15] Jeong Y.S., Jung H.K., Cho K.H., Youn K.S., Hong J.H. (2011). Anti-obesity Effect of Grape Skin Extract in 3T3-L1 Adipocytes. Food Sci. Biotechnol..

[16] Livak K.J., Schmittgen T.D. (2001). Analysis of relative gene expression data using real-time quantitative PCR and the 2(−∆∆C(T)) method. Methods.

[17] Gehart H., Kumpf S., Ittner A., Ricci R. (2010). MAPK signaling in cellular metabolis: Stress or wellness?. EMBO Rep..

[18] Jang Y.J., Koo H.J., Sohn E.H., Kang S.C., Rhee D.K., Pyo S.N. (2015). Theobromine inhibits differentiation of 3T3-L1 cells during the early stage of adipogenesis via AMPK and MAPK signaling pathways. Food Funct..

[19] Chang C.C., Lin K.Y., Peng K.Y., Day Y.J., Hung L.M. (2016). Resveratrol exerts anti-obesity effects in high-fat diet obese mice and displays differential dosage effects on cytotoxicity differentiation, and lipolysis in 3T3-L1 cells. Endor. J..

[20] Kim S.Y., Jang Y.J., Park B.K., Yim J.H., Lee H.K., Rhee D.K., Pyo S.K. (2016). Ramalin inhibits differentiation of 3T3-L1 preadipocytes and suppresses adiposity and body weight in a high-fat diet-fed C57BL/6J mice. Chem. Biol. Interact..

[21] Liu M., Liu H., Xie J., Xu Q., Pan C., Wang J., Wu X., Zheng M., Liu J. (2017). Anti-obesity effects of zeaxanthin on 3T3-L1 pradipocyte and high fat induced obese mice. Food Funct..

[22] Kim J.S., Lee S.G., Min K., Kwon T.K., Kim H.J., Nam J.O. (2018). Eupatilin inhibits adipogenesis through suppression of PPARγ activity in 3T3-L1 cells. Biomed. Pharmacother..

[23] Nagai S., Wakai E., Shibano M., Fujimori K. (2016). Anti-obesity effects of Asian dayflower, commelina communis, in mice with high-fat diet-induced obesity and in 3T3-L1 cells. J. Funct. Foods.

[24] Tang Q.Q., Lane M.D. (1999). Activation and centromeric localization of CCAAT/enhancer-binding proteins during the mitotic clonal expansion of adipocyte differentiation. Genes Dev..

[25] Gregoire F.M., Smas C.M., Sul H.S. (1998). Understanding adipocyte differentiation. Physicol. Rev..

[26] Patel Y.M., Lane M.D. (2000). Mitotic clonal expansion during preadipocyte differentiation: calpain-mediated turnover of p27. J. Biol. Chem..

[27] Poudel B., Lim S.W., Ki H.H., Nepali S., Lee Y.M., Kim D.K. (2014). Dioscin inhibits adipogenesis through the AMPK/ MAPK pathway in 3T3-L1 cells and modulates fat accumulation in obese mice. Int. J. Mol. Med..

[28] Lee H.W., Rhee D.K., Kim B.O., Pyo S. (2018). Inhibitory effect of sinigrin on adipocyte differentiation in 3T3-L1 cells: Involvement of AMPK and MAPK pathways. Biomed. Pharmacother..

[29] Tang Q.Q., Otto T.C., Lane M.D. (2003). Mitotic clonal expansion: a synchronous process required for adipogenesis. Proc. Natl. Acad. Sci. USA.

